# miFRame: analysis and visualization of miRNA sequencing data in neurological disorders

**DOI:** 10.1186/s12967-015-0594-x

**Published:** 2015-07-14

**Authors:** Christina Backes, Jan Haas, Petra Leidinger, Karen Frese, Thomas Großmann, Klemens Ruprecht, Benjamin Meder, Eckart Meese, Andreas Keller

**Affiliations:** Chair for Clinical Computational Biology, Saarland University, Saarbrücken, Germany; Internal Medicine III, Heidelberg University, Heidelberg, Germany; DZHK (German Centre for Cardiovascular Research), Heidelberg, Germany; Department of Human Genetics, Saarland University, Saarbrücken, Germany; Neuroinflammation, Charitee, Berlin, Germany

**Keywords:** miRNA, Alzheimer, Web service, Biomarkers

## Abstract

**Background:**

While in the past decades nucleic acid analysis has been predominantly carried out using quantitative low- and high-throughput approaches such as qRT-PCR and microarray technology, next-generation sequencing (NGS) with its single base resolution is now frequently applied in DNA and RNA testing. Especially for small non-coding RNAs such as microRNAs there is a need for analysis and visualization tools that facilitate interpretation of the results also for clinicians.

**Methods:**

We developed miFRame, which supports the analysis of human small RNA NGS data. Our tool carries out different data analyses for known as well as predicted novel mature microRNAs from known precursors and presents the results in a well interpretable manner. Analyses include among others expression analysis of precursors and mature miRNAs, detection of novel precursors and detection of potential iso-microRNAs. Aggregation of results from different users moreover allows for evaluation whether remarkable results, such as novel mature miRNAs, are indeed specific for the respective experimental set-up or are frequently detected across a broad range of experiments.

**Results:**

We demonstrate the capabilities of miFRame, which is freely available at http://www.ccb.uni-saarland.de/miframe on two studies, circulating biomarker screening for Multiple Sclerosis (cohort includes clinically isolated syndrome, relapse remitting MS, matched controls) as well as Alzheimer Disease (cohort includes Alzheimer Disease, Mild Cognitive Impairment, matched controls). Here, our tool allowed for an improved biomarker discovery by identifying likely false positive marker candidates.

**Electronic supplementary material:**

The online version of this article (doi:10.1186/s12967-015-0594-x) contains supplementary material, which is available to authorized users.

## Background

During the past three decades, molecular analysis of DNA and RNA became more and more important. In the 1990’s, the low-throughput technologies such as Western Blot and quantitative polymerase chain reaction have been augmented by high-throughput technologies, namely microarrays. While microarrays allowed for profiling of thousands of molecules in parallel, producing orders of magnitude more data, qRT-PCR still remained gold standard. All these technologies are thought for quantitative analysis of molecular markers. Around one decade ago, next-generation sequencing (NGS) became available. With its single base resolution, NGS outperformed microarrays by several orders of magnitude considering the information content. This incredible flood of data requires sophisticated computational approaches for quantitative but also qualitative analysis of NGS data. Especially for DNA and mRNA a magnitude of analysis and visualization toolkits, web based as well as stand alone, are available. For miRNAs, that are known to be key regulators and valuable biomarker candidates for various human pathologies, respective tools are currently still under development.

One key challenge in small RNA sequencing analysis is the detection of so far unknown molecules. To address this topic, several tools have been developed. A prominent example is miRExpress, a stand-alone application for detecting known miRNAs along with novel miRNA candidates [1]. Another example, miRanalyzer is available as a stand-alone solution, however it offers also the option to carry out the analyses online in a web based version [2, 3]. Besides the detection of novel miRNAs, miRanalyzer identifies differentially expressed miRNAs and predicts miRNA targets. Likewise as stand-alone and web service, SeqBuster allows for detection of variants of miRNAs for known markers and can moreover be applied to discover dys-regulated miRNAs and the prediction of targets [4].

Besides these tools specialized algorithms such as mirTools [5, 6], DARIO [7], WapRNA [8], eRNA [9] or E-miR [10] have been proposed. Among the most widely applied miRNA NGS tools is miRDeep/miRDeep2, allowing for the prediction of novel miRNAs [11, 12]. Respective tools are now stitched together in pipelines. An example of a comprehensive set of analysis tools is CPSS, including analysis of length distribution and genome mapping, quantification, prediction of novel miRNAs, identification of differentially expressed miRNAs, and functional enrichment, e.g. of GO terms [13]. A second example is omiRas, detecting dys-regulated miRNAs and revealing insights into molecular mechanisms by annotation, comparison and visualization of interaction networks of ncRNAs [14].

While the above mentioned tools that represent just a selection of available NGS analysis solutions, focus on prediction of miRNAs and complex analyses to put miRNAs into a larger context, solutions that specifically analyze and visualize reads across the precursor molecules of specific biomarkers are not common. One example towards this direction is MISIS, a tool to visualize and analyze maps of small RNAs derived from viruses [15]. MISIS displays RNA reads as histogram along a given reference sequence.

However, especially for small non-coding RNAs the visual inspection of reads across the precursor can support the discovery of potentially false positive biomarker candidates, to select the right candidates for further experiments and to interpret the results of the high-throughput experiments. We developed miFRame, a tool for the analysis of single miRNAs as well as miRNA sets from human NGS data that can be easily applied by researchers. Comparable to other tools, miFRame performs quantification of expression on miRNA precursors and mature forms of miRNAs, and allows for discovering differentially expressed markers. Besides this functionality, miFRame however also offers to discover potentially dys-regulated novel mature miRNAs, differentially regulated iso-miRNAs and performs a per-base expression analysis. Moreover, miFRame offers an aggregation analysis. Here, results of one user are compared to other users’ results, e.g., to provide evidence whether a novel miRNA isoform is actually specific for an experiment or frequently detected in other NGS analyses. If this is the case, an NGS artifact may be more likely than an actual finding, allowing for improved priorization of replication/validation experiments. Among the most important features of miFRame besides easy data input and sophisticated analyses along miRNA precursors is a concise representation of results: for each miRNA separately, key findings are represented in various manners, enabling users to inspect the most important markers visually.

## Methods

### miFRame

miFRame is a freely available web-service for analyzing miRNA NGS data (http://www.ccb.uni-saarland.de/miframe/). The front-end is implemented in PHP and substantial parts of secondary miRNA analysis are carried out using proprietary R and Python scripts. No additional packages beyond the core packages in R version 3.0.2 were used. The analyses implemented in miFRame are described in the Results section of the manuscript. miFrame relies on the miRBase (http://www.mirbase.org/), one of the most frequently used miRNA repositories. From the miRBase, the hsa.gff file containing the genomic coordinates for all human miRNAs and the mature.fa file containing all mature miRNA sequences (for all organisms) are used. Currently, miFRame is restricted to human data, thus all non-human miRNAs are filtered out of the analysis. A detailed step-by-step tutorial is available from the web resource.

### Data sets

To demonstrate the functionality of miFRame, we applied our tool to two data sets on Multiple Sclerosis and Alzheimer consisting of a total of 133 samples [16, 17]. In brief, the following cohorts were included: Alzheimer Disease (AD) patients (n = 54), Mild Cognitive Impairment (MCI) patients (n = 20), Alzheimer control set (n = 22), Multiple Sclerosis patients (n = 15), Multiple Sclerosis control set (n = 22). All samples were sequenced on Illumina HiSeq systems. As source, whole blood has been used. Details on library preparation and sequencing are presented in [16, 17]. The data that have been used for this analysis are available for download from the web resource. Beyond the standard parameters we also tested the influence of different parameter sets on the results, specifically the influence of the window sizes is described in detail in the Results section. The pre-calculated examples (Alzheimer and MS) are available from the miFRame homepage without password protection.

## Results

**Figure 1:**
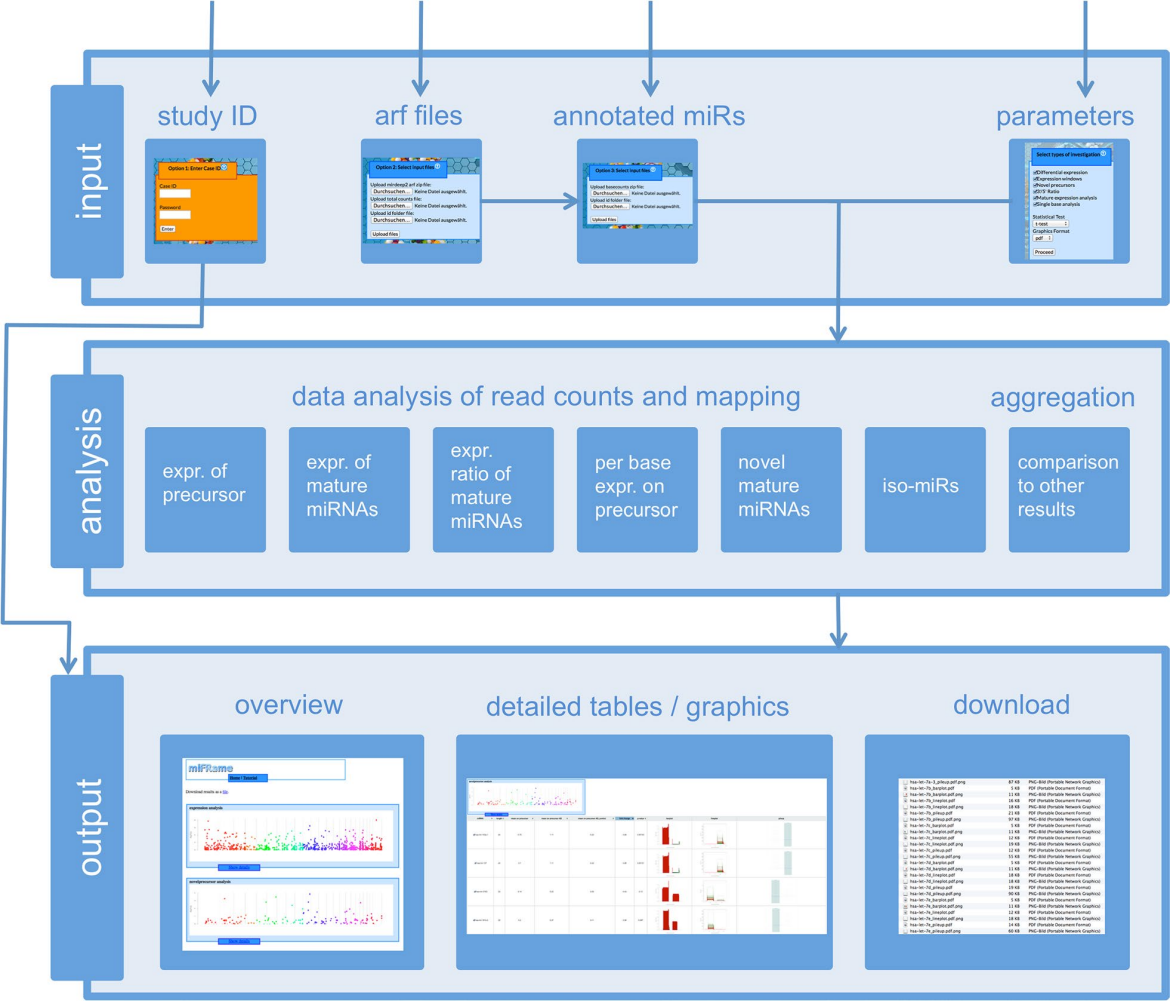
miFRame workflow. The figure sketches the three-layered workflow of miFRame. First, NGS data have to be uploaded along with a parameter set, next statistical analyses are carried out and finally, results are presented to the user.

We developed miFRame, a tool for the analysis and visualization of human miRNA NGS data. miFRame is a web-service developed with the intention to be used by life scientists and clinicians. In this section we will first explain the different input methods for our tool, the available analyses and tested hypothesis and the output. The full workflow is presented in Figure 1. Then we demonstrate the capabilities of miFRame on two NGS data sets. Specifically, the program has been applied in order to investigate miRNAs in Alzheimer and Multiple Sclerosis patients. To this end we collected NGS data from Multiple Sclerosis (MS) patients and respective controls as well as Alzheimer Disease (AD), Mild Cognitive Impairment patients (MCI) and a respective control set. The NGS data have been previously published (see “Methods” section). Altogether, 133 individuals have been included in the analysis and 1,319 miRNAs have been discovered in at least a single sample. Since different NGS runs can yield substantially varying total read counts, all data have been normalized to one million reads (reads per million, RPM).

### Data input

miFRame gets pre-processed NGS data along with a parameter set as input. The parameter input can be easily performed via the web-interface and the following three input options are supported. First, our tool offers to re-enter a previous study ID to access analysis results that have been performed earlier. In that, miFRame also allows for sharing analysis results with collaborators. Besides the unique study ID users also get a password to protect their data. With that ID and the password, collaboration partners can directly access the analysis results (option 1). Second, arf files that are the standard output of miRDeep2, a popular miRNA analysis tool, can be uploaded (option 2). Besides the arf files, the user has to provide a file containing the total number of reads per sample that should be used for normalization. Here, the total reads from the NGS run directly can be used, or alternatively, the read counts after matching to the genome of *H. sapiens* or no normalization can be performed. As third input set, the grouping of the arf file to classes has to be uploaded as tab delimited data file (file name and class for each sample in an own row). Example input is provided for all three files on the miFrame homepage. Third, it is possible to use any other miRNA annotation tool and to generate the annotation files for upload with miFRame (option 3). The latter option allows for largest flexibility and transfer of small data sets in the range of few megabytes but substantial local pre-processing by the user is required. A more detailed description of the different input formats is available on the miFRame main webpage (http://www.ccb.uni-saarland.de/miframe/).

As parameters the user can specify significance threshold, select whether just single positions or many bases across the precursor have to be significantly changed (to observe differential regulation of one mature miRNA, around 15 positions should at least be significant), a window size for detection of iso-microRNAs (typical parameters are 2–4 bases) and the different analyses to be carried out. Moreover, the user can specify the statistical test to be applied (parametric *t* test for normally distributed data or Wilcoxon Mann–Whitney test) and the graphics output format (pdf vector graphics, jpeg or png). Finally, the user can decide whether the data should be used for the aggregation analysis. In this case he gets the number of significant results others obtained for the respective miRNAs and his own data are added to the data pool.

### Analysis

#### Expression analysis

Here, miFRame tests the hypothesis that all reads mapping to the precursor are differentially expressed between a case and control cohort. The mean read count per sample across the precursor is calculated. By applying the hypothesis test specified by the user, average count in case and control cohort is compared to each other and a p-value per miRNA precursor is calculated.

#### Mature expression analysis

In this step, miFRame tests the hypothesis that miRNA precursors are differentially expressed between cases and controls. To this end, the average read count per sample on each mature 3′ and 5′ miRNA is calculated and by applying the hypothesis test specified by the user, a p-value is calculated.

#### 3′ to 5′ expression analysis ratio

Here, miFRame searches for miRNAs that show either opposite or same differential expression between cases and controls on the 3′ compared to the 5′ mature forms of miRNAs. First, miRNAs with just a single mature form are omitted, first. Then, for each remaining miRNA the fold changes and p-values for the 3′ and 5′ forms are determined analogously to the mature expression analysis. From these two fold changes, the difference between the 3′ and the 5′ mature form is calculated. miRNA precursors with resulting expression quotients close to 1 show the same up- or down-regulation of both mature forms, while those miRNAs with fold changes ≫1 are more up-regulated in the 5′ mature form and miRNAs with fold changes ≪1 are more up-regulated in the 3′ mature form.

#### Per-base expression analysis

In this analysis, miFRame checks how many base positions across the precursor of each miRNA are significantly differentially expressed in cases compared to controls. Most computational approaches just consider either maximal or average expression across the mature forms of miRNAs. In several cases, however, just few positions, especially at the 3′ or the 5′ end of the mature miRNA(s) are significantly different between two cohorts. These sites are detected by the per-base expression analysis but are usually not discovered by considering the full precursor or mature miRNAs (see also window analysis).

#### Novel mature miRNA analysis

For a substantial amount of miRNAs in the miRBase just a single mature form (either the 3′ or 5′) is known. However, in many cases a significant number of reads maps exactly to positions matching the respective second mature form (5′ or 3′) of known miRNAs. To this end, miFRame calculates for those miRNAs where just a single mature form is known whether reads are matching to the respective 3′ or 5′ mature form. Additionally, miFRame calculates whether the respective mature form shows significantly different expression in cases as compared to controls and outputs how many reads are located on this potential novel mature miRNA.

#### Window analysis

As described in the per base analysis section, it is often the case that only a few single positions, predominantly at the 3′ or 5′ end of the mature miRNA(s) belonging to one precursor are significantly changed or show at least a lower p-value value than the remaining part of the precursor or mature form(s) of the miRNA. These sites are frequently not taken into account in statistical analyses or are down-weighted by averaging across the mature miRNAs. miFRame allows the user to select a window size in the parameter selection step. Then, it calculates the percentage of bases covered in this window with respect to the 3′ and 5′ end of the 3′ and 5′ mature miRNA form. In consequence, for each precursor with two mature forms 4 percentages in cases and controls are calculated along with the difference between the two cohorts while for precursors with just one mature form only two percentages are calculated. Additionally, miFRame also outputs the respective motifs at the 3′ and 5′ end of the mature miRNAs. Thus, mature miRNAs with isoforms in specific traits can be well detected.

#### Aggregation analysis

In case the user decided to carry out the aggregation analysis, just the relevant findings from the current study are stored anonymously in a local database. In return, the relevant findings from that user are compared to the database such that it becomes clear whether e.g. a novel mature miRNA is specific for a certain trait or is reported in many experiments, making a false positive finding by NGS artifacts more likely. This analysis may help to prioritize the replication and validation experiments by selecting findings that are less frequently discovered.

### Output of results

**Figure 2:**
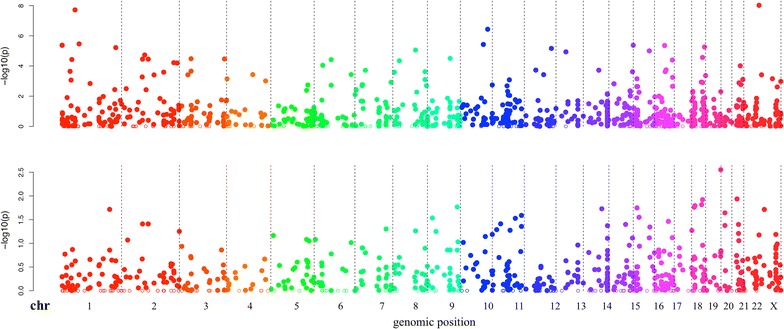
Manhattan plots. The results of the expression analysis for MS and AD are presented. Each microRNA included in the set is located on the genome (x-axis) and for this miRNA the negative decade logarithm is presented on the y-axis. The higher the respective data points, the more significant the results are. The graphic reveals that the AD study showed increased significance as compared to the MS study (scale on the y-axis).

One key criterion for miFRame was the concise graphical representation of results such that the high throughput data can be explored in great detail by researchers in order to detect potential false positive mappings. Our tool offers three different output categories. First, all relevant results per analysis are shown as Manhattan plots. These plots, well known from genome wide association studies (GWAS) show the negative decade logarithm of p-values as function of the genomic position, allowing for detection of hot spots in the genome immediately. Two examples for such Manhattan plots, representing the results of the expression analysis for Alzheimer versus matched controls and Multiple Sclerosis patients versus matched controls, are presented in Figure 2.

As second output, miFRame presents detailed results for all analyses that lead to significant findings. The result tables consist of two parts, the left hand contains key characteristics such as read counts, p-values, fold changes, etc. All entries can be sorted in increasing or decreasing manner for the respective features. The right hand part of the result tables consists of three thumbnails for each miRNA. Clicking each of them opens high-resolution graphical representations of the respective results. The three representations include the following plot: (1) significant bases across the precursor molecules. For each miRNA, the negative decade logarithm of p-values is presented. Remarkably, for each base of the precursor an own bar is drawn. Green bars represent down-regulation in cases while red bars mean up-regulation in cases as compared to controls. (2) read distribution across the precursor. Each sample is presented as line plot along the miRNA precursors. Here, red lines correspond to cases while green lines correspond to controls. (3) Pileup plots for each miRNA are also calculated. These plots show in the middle the precursor miRNA sequence. Mature forms are colored in blue and red, respectively, and the area where reads map to known miRNAs is shaded in blue. Above the precursor sequence, the consensus sequence per case individual is presented while below the precursor sequence the consensus per control individual is drawn. Interpreting these results allows for easy detection of miRNAs that show inaccurate mappings, indicating potentially false positive findings, or for discovering iso-miRNAs. In case the user decided to carry out the aggregation analysis, a fourth plot is generated, showing in how many different experiments the respective miRNA has been included and in how many of these experiments a significant finding was calculated.

Besides the web-based representation of the results all findings can be downloaded as compressed folder. The download does not include just the Manhattan plots, bar diagrams, read distribution graphics and pileup plots but also tab delimited flat files that can be imported in various standard software for spreadsheet analysis.

### Application of miFRame to Alzheimer and multiple sclerosis

To demonstrate the usability of miFRame we applied the tool to two publicly available data sets that can be also downloaded from the miFrame homepage, containing circulating miRNA profiles from whole blood of Alzheimer and Multiple Sclerosis patients [16, 17]. The data sets are sketched in the “Methods” section. Detailed description can be found in the respective original publications. For both data sets the full analysis has been carried out using the standard parameters of miFRame and analysis results are available from the miFRame homepage (reference ID and password are provided in the “Methods” section). All analyses have been carried out on samples with read counts normalized to one million total reads.

#### Expression analysis

**Figure 3:**
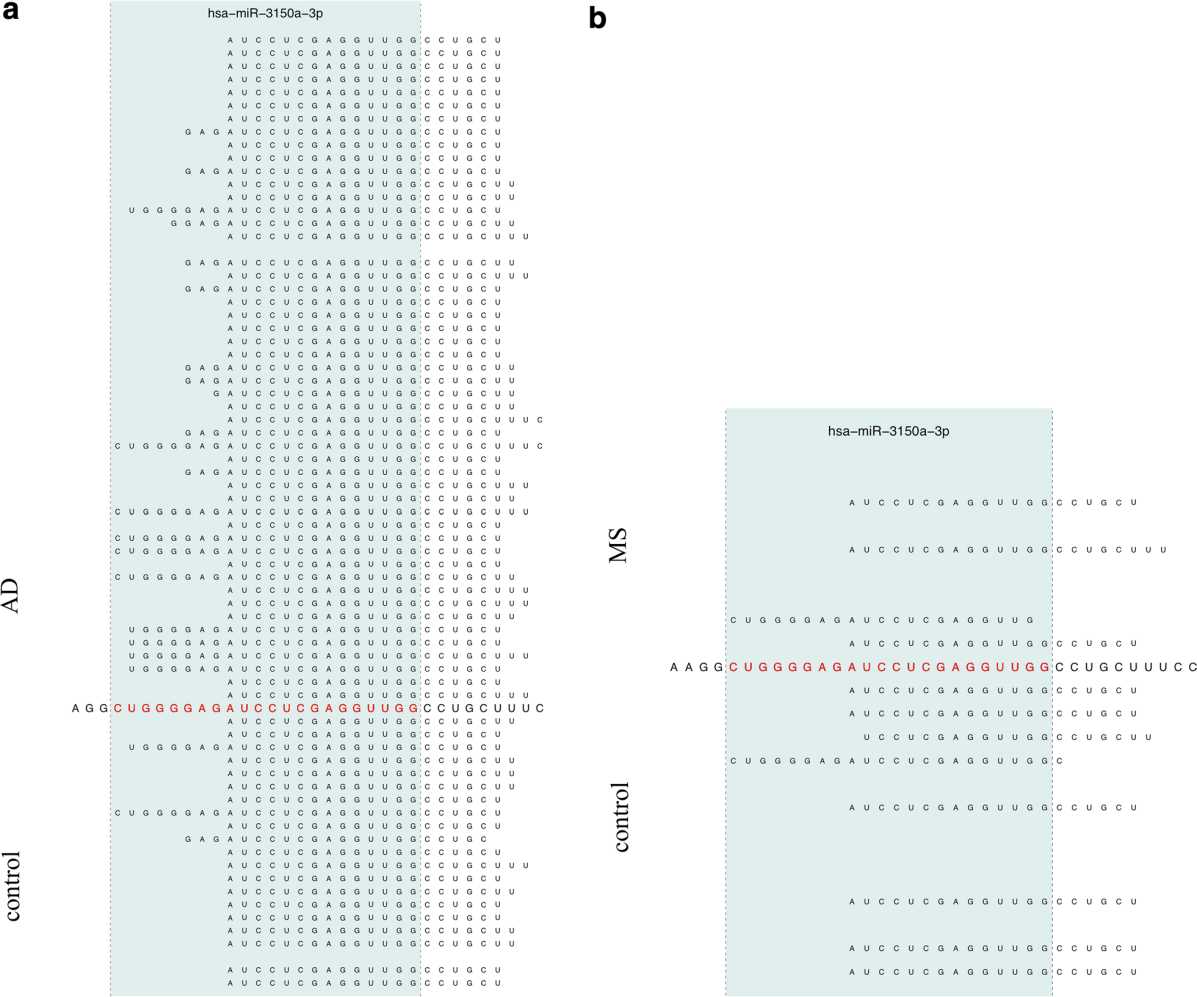
Pileup plot for miR-3150a-3p. The figure presents an extract of pileup plots for hsa-miR-3150a-3p in the two studies (**a** AD; **b** MS). Samples on *top* represent cases and on *bottom* controls. Each sample where at least a single read mapped to the precursor get an own *line*. The sequence in the middle corresponds to the precursor sequence, the mature miRNA (in this case the 3p mature form) is colored in *red*. The *blue shaded area* marks the mature part of this miRNA. In both cases, AD and MS, reads are partially mapping outside the mature form of this miRNA.

In Figure 2, Manhattan plots for the expression analysis of both data sets are presented. The Alzheimer panel on top of the figure reveals substantially more dys-regulated miRNAs on chromosome 1 and 2 (left part) as compared to the Multiple Sclerosis data set on bottom of the figure. Vice versa, the Multiple Sclerosis data set shows a tendency for clustering of dys-regulated miRNAs on the smaller chromosomes (right part of the figure). Altogether, the Alzheimer data set shows much lower p-values and thus by far more significantly dys-regulated miRNAs. The most significant dys-regulation in case of Alzheimer was calculated for hsa-miR-1468-5p (p = 9 × 10^−9^) while the most significant miRNA in case of Multiple Sclerosis was hsa-miR-3179 (p = 0.001). In both cases the visual inspection of the mapping results did not reveal critical read alignments but the NGS reads mapped well to the known mature miRNAs. Another miRNA, which was significant in Alzheimer Disease, was hsa-miR-3150a-3p (p = 6 × 10^−5^, not significant in Multiple Sclerosis). Here, the pileup plots, however, showed a substantial number of reads mapping outside the actual mature form of the miRNA in Alzheimer and Multiple Sclerosis patients as well as matched controls (Alzheimer vs. controls: Figure 3a, Multiple Sclerosis vs. controls Figure 3b). Since, driven by the larger cohort size, the Alzheimer panel showed more significant results as compared to the Multiple Sclerosis NGS study, we focus in the following on Alzheimer Disease.

**Figure 4:**
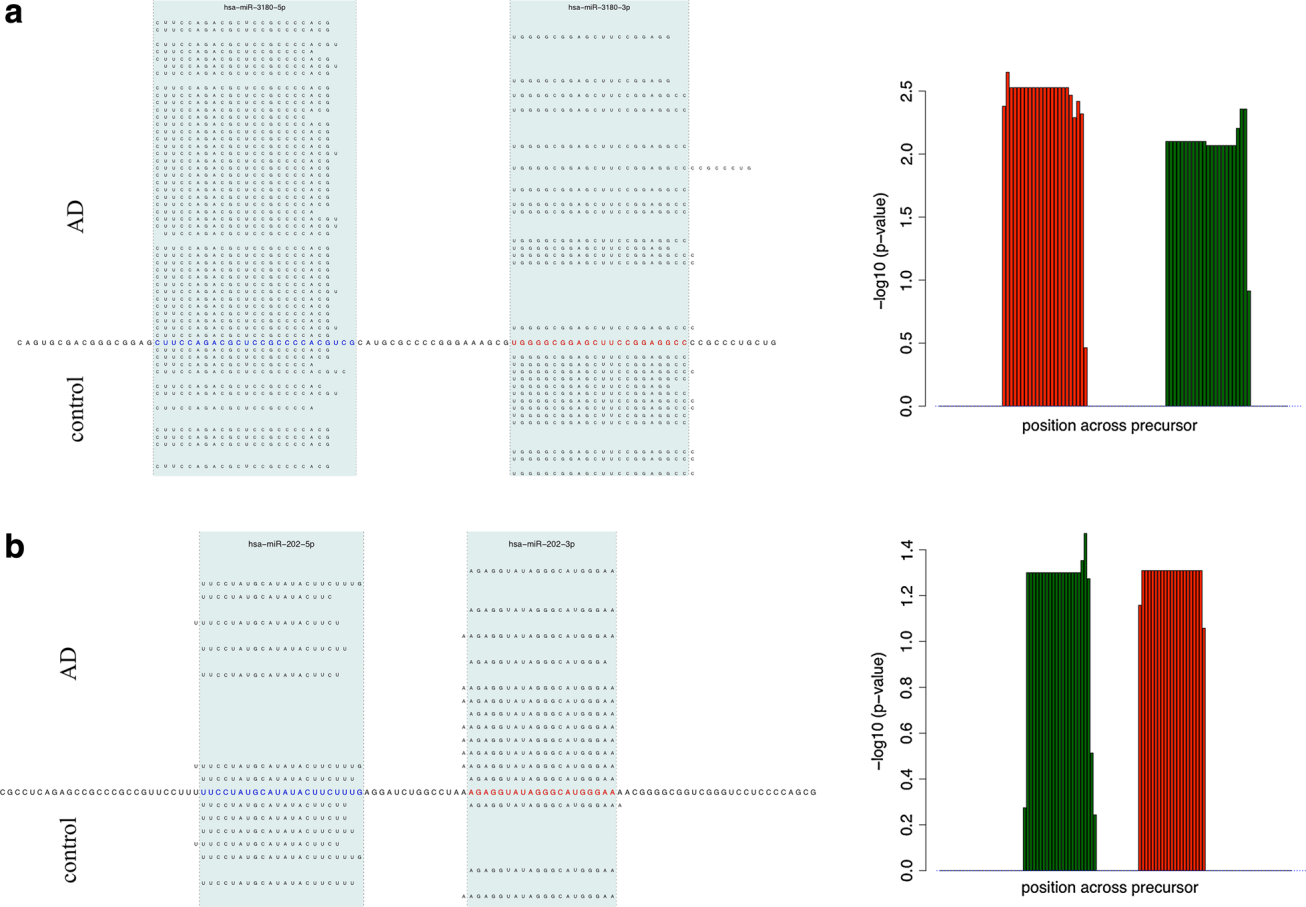
Expression ratio of 3p and 5p mature forms. In this figure, opposite fold changes of the 3p and the 5p mature form of miRNAs is shown for two miRNAs, miR-3180 in panel **a** and miR-202 in panel **b**. The *right part* shows the pileup plots, the sequence in the middle represents the precursor, the two mature forms are highlighted in *red* (5p) and *blue* (3p) and with the *blue shaded area*. The right hand side shows the negative decade logarithm of the p-value for each single position. Up-regulated positions on the precursor in controls are colored in green and red bars correspond tow don-regulated positions on the precursor. The two *red* and *green* blocks correspond to the two mature forms, respectively. Clearly, the 3p form is up-regulated while the 5p form is down regulated for miR-3180 (*panel* **a**) while miR-202 shows the opposite behavior (*panel* **b**).

As next step following the expression analysis we specifically searched for miRNAs where both mature forms show significant dys-regulation. Indeed we detected several miRNAs with up-regulation of the 3′ form and down-regulation of the 5′ mature miRNA or the opposite behavior. Remarkably, miR-3180 showed up-regulation of miR-3180-3p (p = 0.007) while miR-3180-5p was significantly down-regulated (p = 0.003). Here, the degree of differential expression between the 3′ and the 5′ form was 7-fold. Vice versa, miR-202 showed down-regulation of miR-202-3p (p = 0.05) while miR-202-5p was significantly up-regulated (p = 0.04). In this case, the degree of differential expression between the 5′ and the 3′ form was again sevenfold. Pileup plots for both miRNAs showing the accurate mapping pattern of reads to the mature miRNAs are presented in Figure 4 (a: miR-3180, b: miR-202).

While these analyses generally could have been done using qRT-PCR or microarray data, the NGS data however offer the additional avenue of a detailed per base data analysis.

#### Analysis of mapped reads

**Figure 5:**
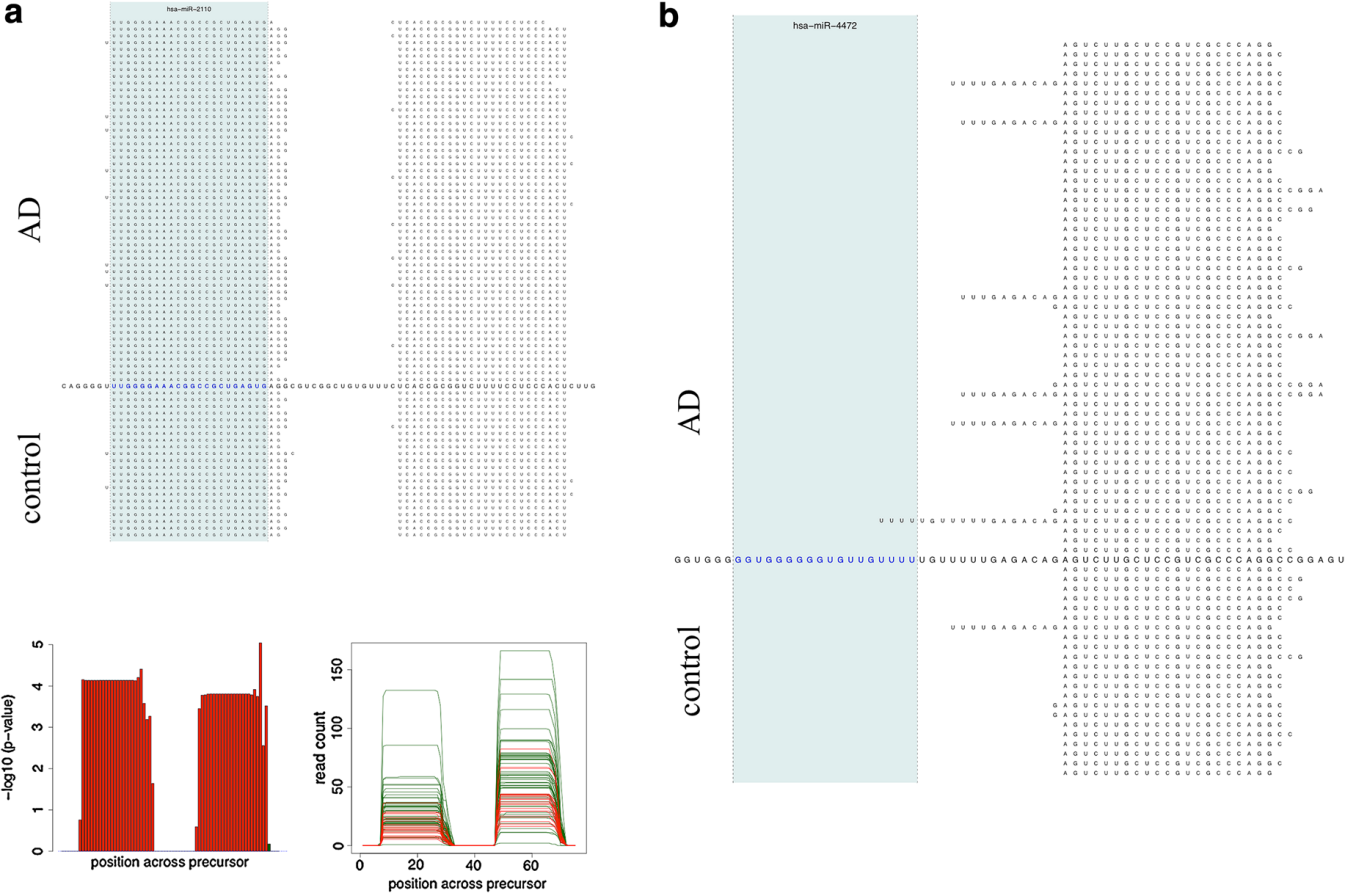
**a** Potential novel mature miRNA. The *upper part* of the figure presents the pileup plot (similar to Figures 3, 4). Just one mature form is annotated in miRBase V20, namely the 5p form (*colored* in *red* and shaded in *blue*). Nevertheless, many reads map to the position where a 3p version could be located. The lower right part shows the significant down-regulation of this miRNA in controls as compared to Alzheimer Disease patients. The *lower left* image presents beyond the significance the absolute rad count per sample. *Green samples* correspond to cases and red samples to controls. **b** For miR-4472, just one mature form (5p) is annotated in the miRBase. While we do not observe mappings to this position, we observe mappings to the position where the 3p mature form could be expected.

First, we investigated whether we find reads mapping to precursors where just the 3′ or the 5′ mature miRNA has been annotated in miRBase 20, whether the respective reads are mapping to the potential novel mature miRNA in an adequate manner and whether the respective potential novel mature miRNA is dys-regulated between Alzheimer Disease patients and matched controls. Indeed, we found several miRNAs matching the criteria above. One of the most prominent examples was miR-2110. Here, we found a potential novel mature form consisting of 26 bases. On average, 52 reads mapped to this novel mature form, more interestingly, expression for Alzheimer Disease patients was 1.6 fold higher than for controls (p = 0.0001). The graphical output generated by miFrame for this miRNA is presented in Figure 5a (upper part: pileup plot, lower right part: along precursor read distribution, lower left part: along precursor p-values). While this mature form has not been annotated in the miRBase, yet, a recent publication described the respective miRNA as novel mature 3′ form of miR-2110 [18]. In this case, the aggregation analysis highlighted that in so far 100% of all analyses (time of analysis: June 2015) carried out whit the aggregation option enabled this novel mature miRNA was observed. In some cases, we observe miRNAs in a position of a precursor where no mature form is expected at all while the actual mature form as observed in the miRBase is not covered by a single read. One respective example, miR-4472-2 is presented in Figure 5b. Again, reads outside of the annotated 5′ mature miRNA were observed in 100% of all analyses carried out.

**Figure 6:**
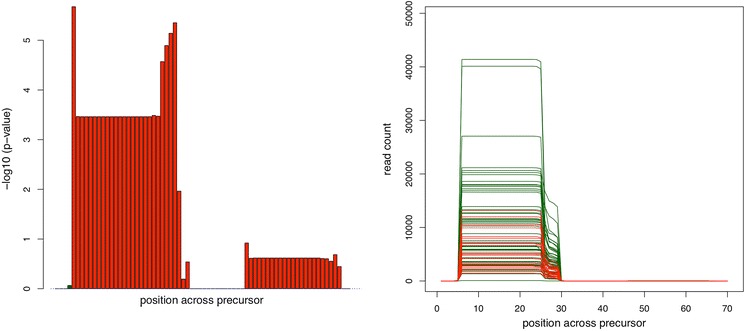
potential isoforms of miR-30d-5p. The bar-plot on the right hand side shows increased significance at the edges of miR-30d-5p. In this case, Alzheimer Disease patients have higher expression on few base positions outside the annotated miRNA. This can be also seen on the read distribution plot on the right hand side. At position 26–29, Alzheimer Disease patients (*green*) have significantly higher coverage than controls.

**Figure 7:**
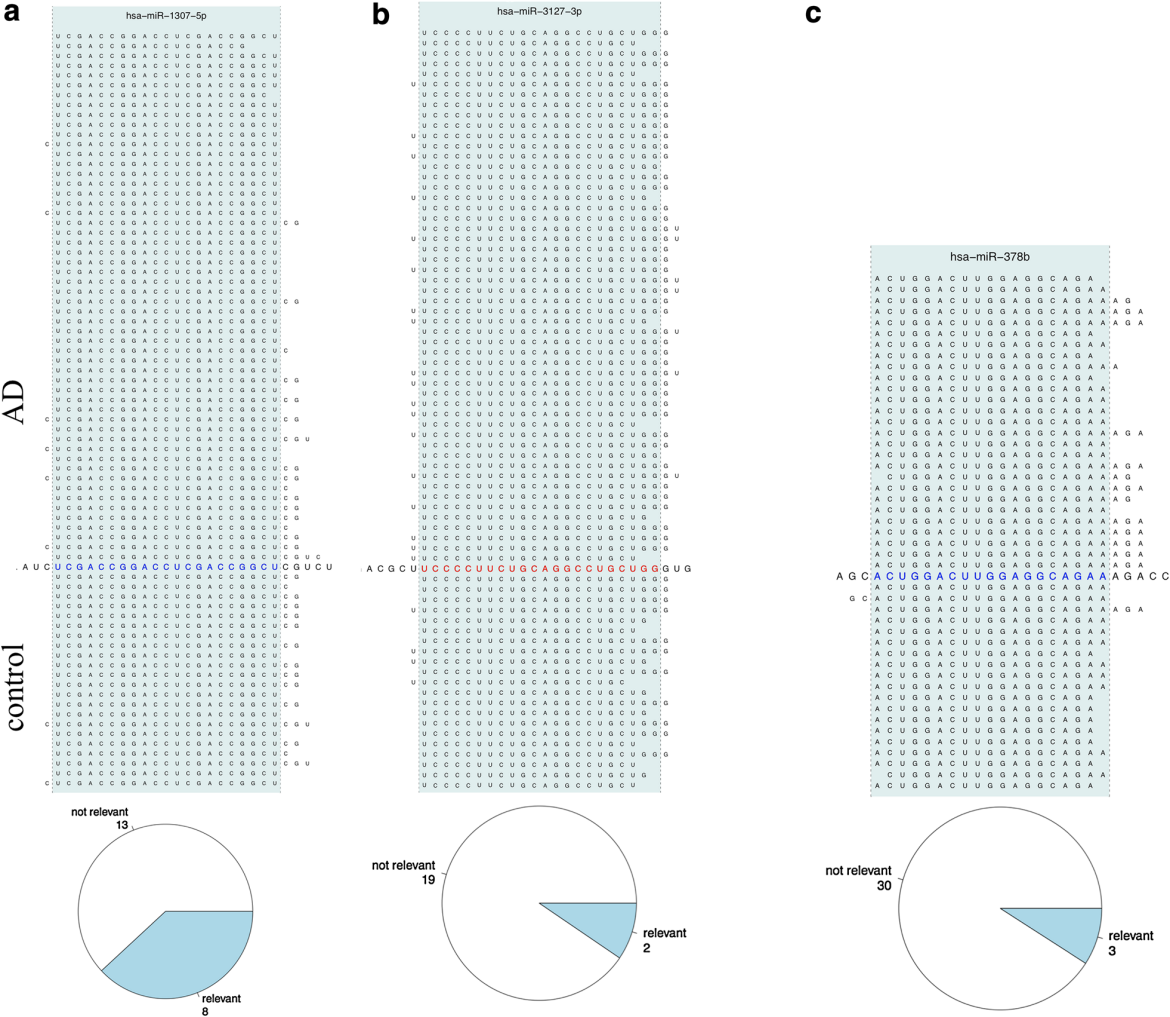
**a** Pileup plot for miR-1307. In case of miR-1307-5p, control patients show increased frequency of 1–2 base shifts at the 3p end. The *pie diagram* at the *lower part* of the figure shows that around one-third of all analyses revealed respective iso-forms. **b** Pileup plot for miR-3172. For miR-3127-3p, AD samples are frequently shifted by 1–2 bases to the 3p end of this miRNA. The *pie chart* as result of the aggregation analysis indicates that respective results are usually not observed. **c** Pileup plot for miR-378b. Extended mappings at the 3p end of this miRNA are almost exclusively observed for AD patients.

Besides detecting novel mature miRNAs, miFRame also offers to discover iso-miRNAs. Here, specifically variants with extended mature miRNA reads in a window specified by the user are reported. To quantify the effect, miFRame calculates the frequency of reads mapping for samples in the window outside the annotated miRNA. Besides the tabular output, again, the respective graphical output supports to detect potential iso-microRNA candidates. These are shown by peaks in the significance plots at the 3′ and/or 5′ end of known mature miRNAs. A representative example is presented in Figure 6. Here, the significance and read distribution across the precursor for miR-30d is presented. For the 5′ mature form of this miRNA, significantly extended reads in case of Alzheimer Disease patients can be detected. While the average significance is in the range of 2 × 10^−4^, p-values for the 3′ and 5′ end of the mature miRNA are as high as 10^−6^. Other interesting examples are shown in Figure 7. Figure 7a represents the pileup plot for miR-1307-5p. Here, control samples show in 64% of cases an extension by 1 or 2 bases at the 3′ side of the 5′ mature miRNA. In contrast, just 29% of Alzheimer Disease patients show such an extension by 1 or 2 bases. The aggregation analysis showed that respective differences are observed in around one-third of all analyses (pie plot in the lower part of that figure). Figure 7b presents the pileup for miR-3127-3p. In this case, Alzheimer Disease patients show a doubled frequency of bases covered to the 3′ end of this miRNA as compared to controls. Here, the aggregation analysis revealed that respective changes are usually not observed. Finally, Figure 7c presents miR-378b. The 3′ end of the 3′ miRNA is covered in 50% of all cases at a window size of 2 while this holds just for 5% of controls. Again, just very few results had similar reads mapping at the 3′ end of this miRNA.

#### Influence of the window size

Especially the parameter of the window size potentially has a substantial influence of the results of miFRame. We thus explored how divergent results are with respect to different window sizes ranging from 1 to 4. Thereby we observed a generally high stability of results independent of the window size. For the three before mentioned miRNAs, the results are presented in Table 1. miR-1307 has with 1-base window values of 31.5% (AD) and 68.1% (control) with a fold change of 0.46. For 2-base windows values shift to 28.7 and 63.6% (fold change of 0.45). For 3-base windows values are 20.4 and 45.5% (fold change of 0.45) and for 4-base windows 15.7 and 34.4%. Although the percentages in AD and control substantially decrease from 1 to 4 bases, the ratio stays constant. The same holds also for the other miRNAs in Table 1.

**Table 1:** Influence of the window size on the results computation of miFRame

miRNA	1 base	2 base	3 base	4 base
miR-1307				
AD	31.5	28.7	20.4	15.7
Control	68.1	63.6	45.5	34.4
Quotient	0.46	0.45	0.45	0.46
miR-378b				
AD	51.9	50	43.2	32.4
Control	5.3	5.3	5.3	3.9
Quotient	9.8	9.4	8.2	8.3
miR-3127				
AD	82.7	48.1	32.1	24
Control	50	25	16.7	12.5
Quotient	0.6	0.52	0.52	0.52

## Discussion

As described in the Introduction, analysis tools for next generation sequencing data of small non-coding RNAs are constantly improved and novel tools are implemented. While a substantial portion of these tools focuses on the detection of novel miRNAs from NGS reads, other tools aim at improved understanding of pathogenic processes by integrating miRNA data to biochemical pathways and linking them with target genes.

Besides furthering our understanding of human pathogenic processes, miRNAs however offer themselves as biomarkers to detect disease in time or monitor disease progression. With miFRame, we developed a tool that supports the investigation and graphical analysis of biomarker candidates. Besides classical quantitative analyses, miFRame also provides qualitative analyses such as the detection of novel mature forms of miRNAs or the discovery of potential iso-miRNAs, specifically extended mature forms of miRNAs.

Key functionality of miFRame is the concise graphical representation of the high-throughput data. For each analysis, an overview plot showing the significant results along the genome is generated, a well-known representation from Genome Wide Association Studies. More importantly, three plots are generated for each miRNA, showing the significance and respectively read distribution along the miRNA precursor together with pileup plots, representing the consensus sequence of all reads and all samples mapping to this precursor.

The potential of miFRame is presented on Multiple Sclerosis and Alzheimer Disease whole blood NGS data. Beyond the known markers for this disease, especially in case of Alzheimer novel markers were identified. The increased significance in AD compared to MS may be partially due to the larger cohort size for the AD study. Beyond discovering of differentially regulated miRNAs, our analysis also revealed miRNAs with different fold changes on the 3′ to 5′ form in AD as compared to controls, novel potential mature forms of known miRNAs as well as potential isoforms. Remarkably, in case of the Alzheimer data set, we originally reported a 12-miRNA signature that has been fully validated by qRT-PCR. While in 10 cases results matched well, for 2 miRNAs, however, the qRT-PCR data did not correspond to the NGS results (miR-26a-5p and miR-1285-5p). For miR-26a-5p, miFRame did not reveal a potential source leading to a false positive biomarker. In contrast, for miR-1285, miFRame revealed a wired read mapping to the mature 5′ form on the precursor. The results are shown in detail in Additional file 1: Figure S1. Only few bases at the 5′ end of the miRNA were highly significant, the difference between Alzheimer Patients and controls decreased towards the middle of that miRNA and many reads mapped outside the actual annotated mature 5′ biomarker, representing a potential mapping issue in case of this biomarker, which may lead to a divergent qRT-PCR result.

## Conclusion

We developed miFRame, a tool that gets miRNA NGS data and carries out statistical analysis of read counts as well as detection of novel mature miRNAs and iso-miR analysis. Compared to other tools, miFRame has multiple additional features, for example users can share their aggregated and anonymized results and as benefit get the feedback how many other researchers observed similar results, highlighting potential systematic errors and help to prioritize candidates for validation studies. The very detailed graphical representation of the results allows researchers to interpret and evaluate relevant findings from their experiments. Thus, our tool augments current miRNA NGS analysis pipelines that are tailored for the detection of novel miRNAs, the prediction of target genes or functional miRNA enrichment analysis.

## Additional file

**Additional file 1.** Pileup plots for small RNA reads mapping to miR-1285.
