# Supplementary figures and images for: miFRame: analysis and visualization of miRNA sequencing data in neurological disorders
Source: J Transl Med. 2015 Jul 14;13:224. doi: 10.1186/s12967-015-0594-x (PMC4501052; doi:10.1186/s12967-015-0594-x)

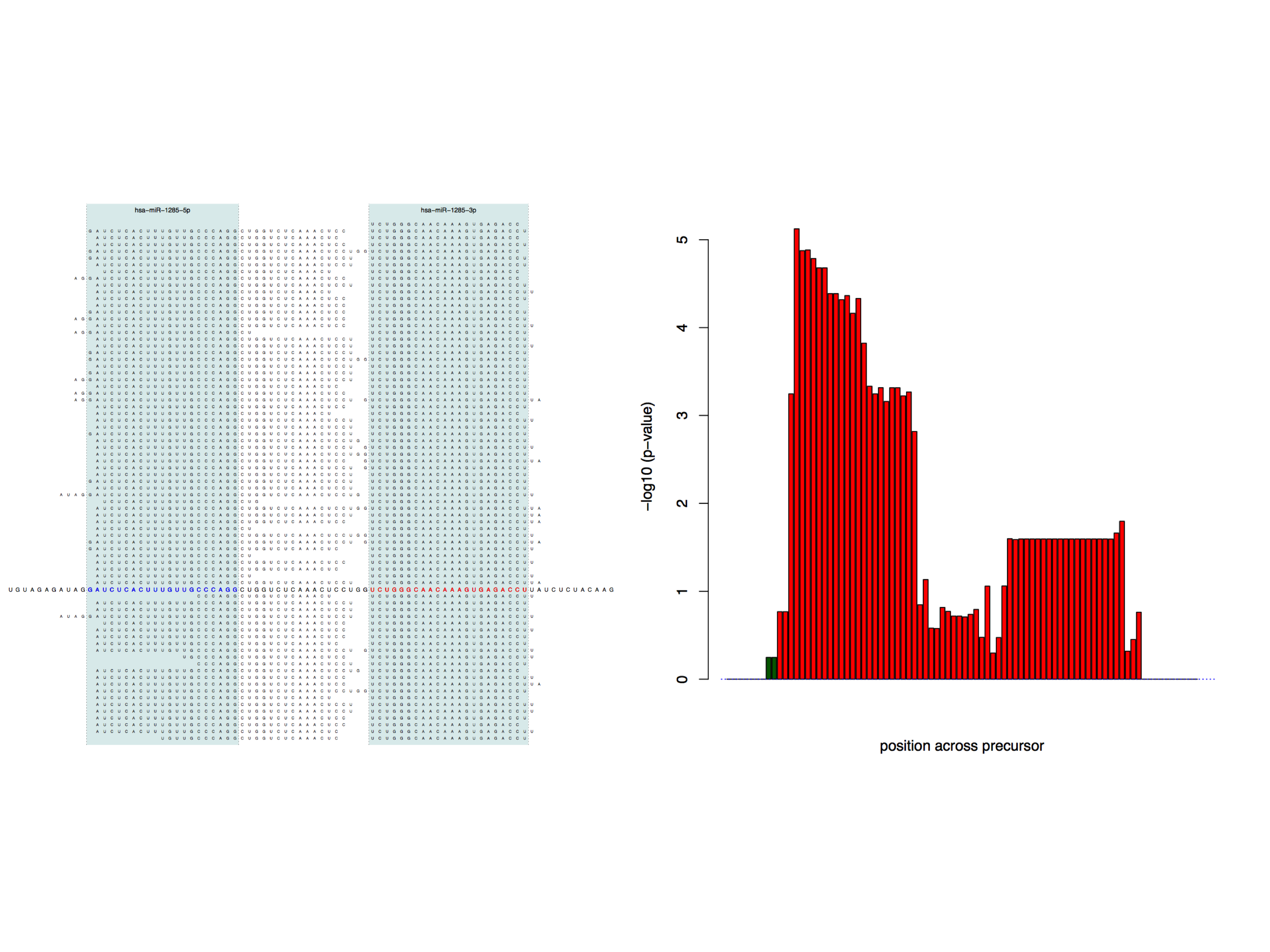

Supplement: Supplementary file 1 — Additional file 1. Pileup plots for small RNA reads mapping to miR-1285. [file 12967_2015_594_MOESM1_ESM.png]
